# Histological variants of cutaneous Kaposi sarcoma

**DOI:** 10.1186/1746-1596-3-31

**Published:** 2008-07-25

**Authors:** Wayne Grayson, Liron Pantanowitz

**Affiliations:** 1 Histopathology Department, Ampath National Laboratory Support Services, Johannesburg, South Africa; 2 Department of Pathology, Baystate Medical Center, Tufts University School of Medicine, Springfield, Massachusetts, USA

## Abstract

This review provides a comprehensive overview of the broad clinicopathologic spectrum of cutaneous Kaposi sarcoma (KS) lesions. Variants discussed include: usual KS lesions associated with disease progression (*i.e. *patch, plaque and nodular stage); morphologic subtypes alluded to in the older literature such as anaplastic and telangiectatic KS, as well as several lymphedematous variants; and numerous recently described variants including hyperkeratotic, keloidal, micronodular, pyogenic granuloma-like, ecchymotic, and intravascular KS. Involuting lesions as a result of treatment related regression are also presented.

## Introduction

Kaposi sarcoma (KS) is a vascular lesion of low-grade malignant potential that presents most frequently with skin lesions. Most histopathologists are *au fait *with the histologic picture of usual (typical) cutaneous KS as it progresses from patch, to plaque and finally nodular stages [1-4]. This morphologic spectrum of "usual" KS is common to classic, African endemic, transplant-associated, and acquired immune deficiency syndrome (AIDS)-associated KS [4]. In recent decades, however, there has been an increasing awareness of a wider histologic spectrum [5,6]. This has resulted in a growing number of reported clinical and/or histologic variants of KS. Failure to identify a given lesion as KS could lead to delayed diagnosis or inappropriate management. It has also been suggested that certain variants, such as anaplastic KS and possibly lymphangioma-like KS, might have prognostic relevance [7-9].

Taking these factors into account, we have elected to divide KS skin lesions into four broad groups: (1) KS lesions which encompass usual variants related to disease progression, (2) KS variants alluded to in the older literature, (3) more recently described KS variants, and (4) KS lesions as a consequence of therapy. Although the apparent histogenesis of some of these morphologic variants is known, the pathogenesis of others is uncertain or subject to speculation. Hyperkeratotic KS, by way of example, frequently occurs as a result of KS-associated chronic lymphedema of the lower extremities [10]. Intravascular KS, on the other hand, could either originate primarily as an intravascular proliferation, or alternatively develop as a consequence of intravascular extension of a lesion breaching the vessel wall [11]. KS lesions arising in extracutaneous locations often differ histologically from their counterparts in the skin, including the recent description of "*in situ*" KS involving mediastinal lymphatic vessels [12].

### Usual variants related to progression

#### Patch stage

**Figure 1:**
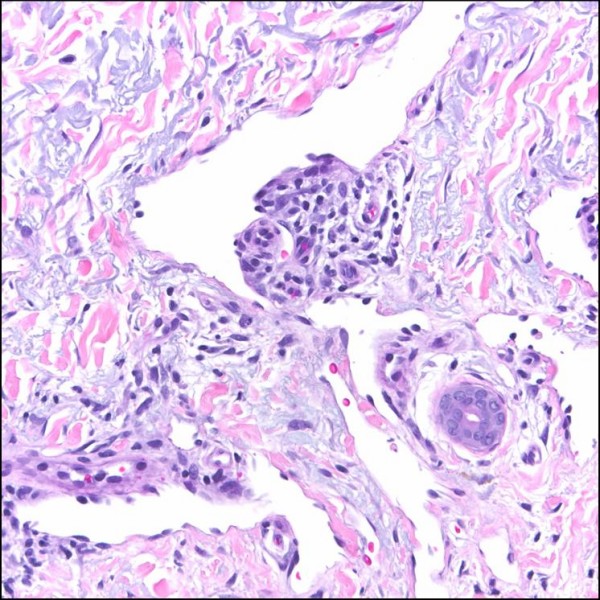
Patch stage Kaposi sarcoma showing newly formed vessels protruding into a larger vascular space characteristic of the promontory sign (H&E stain).

Patch stage KS, which represents the earliest phase in the evolution of cutaneous KS, is perhaps the histologic variant with the greatest propensity to cause diagnostic difficulties for the unwary. The initial low-power impressions are those of a "busy" dermis, or perhaps some form of mild inflammatory dermatosis [1,13]. On closer examination, however, there are signs of a subtle vasoformative process composed of newly formed slit-like or somewhat jagged vascular spaces, which tend to be more conspicuous in the immediate vicinity of native dermal vessels and cutaneous appendages [1-4]. The protrusion of these native microscopic vascular structures into the lumens of more ectatic neoplastic channels results in the characteristic promontory sign (Figure 1). The intervening dermis frequently reveals dissection of its collagen bundles by slit-like vascular spaces lined by a monolayer of relatively banal, flattened endothelial cells, with a variable degree of erythrocyte extravasation. The newly formed channels often contain red blood cells. There is also a noticeable mild background inflammatory cell infiltrate comprising lymphocytes and plasma cells, often accompanied by a contingent of hemosiderin-laden macropahges. The aforementioned mononuclear cells tend to be concentrated around the native vessels and skin adnexal structures [1-4].

#### Plaque stage

**Figure 2:**
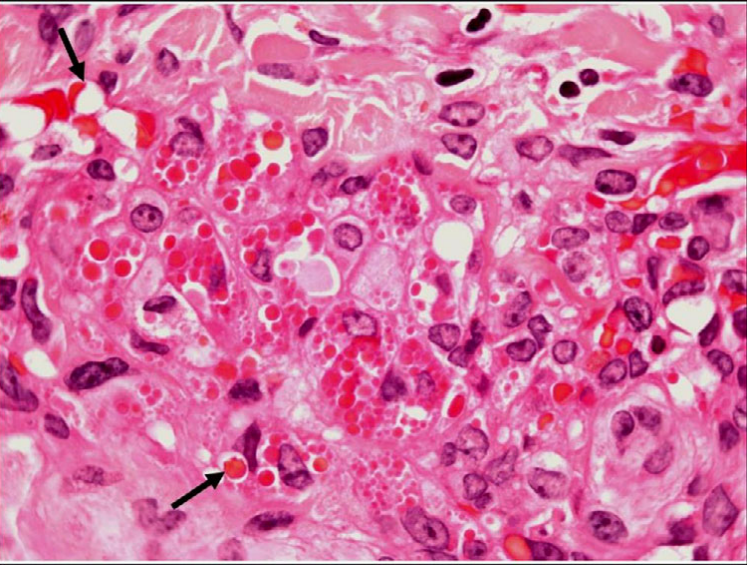
**Plaque stage Kaposi sarcoma**. Large numbers of intracellular and extracellular eosinophilic hyaline globules are visible in this field (H&E stain). The arrows indicate so-called "autolumination", with paranuclear vacuoles containing erythrocytes.

In plaque stage lesions of KS, the histologic picture is characterized by a more diffuse dermal vascular infiltrate, accompanied by greater cellularity and occasional extension of this process into the underlying subcutaneous adipose tissue. The lesional cells tend to be more spindled and arranged in short, sometimes haphazard fascicles [1-4]. Fascicles cut in cross section demonstrate a sieve-like appearance. Mitotic figures are sparse and there is no significant nuclear or cytological pleomorphism. Intra- and extracellular hyaline globules, probably representing effete erythrocytes, are often seen. Careful scrutiny will frequently reveal "autolumination", whereby an erythrocyte is contained within a clear paranuclear vacuole in the cytoplasm of a spindled endothelial cell observed in cross section (Figure 2). Numerous dissecting vascular channels containing erythrocytes occupy the intervening dermis, and once again there is evidence of a background plasma cell-rich contingent of chronic inflammatory cells with admixed siderophages and free-lying hemosiderin pigment. The promontory sign, as described above, may also be encountered [1-4]. The histologic differential diagnosis includes tufted angioma, targetoid hemosiderotic hemangioma, microvenular hemangioma and acroangiodermatitis ("pseudo-Kaposi's sarcoma") [1,2,4].

**Figure 3:**
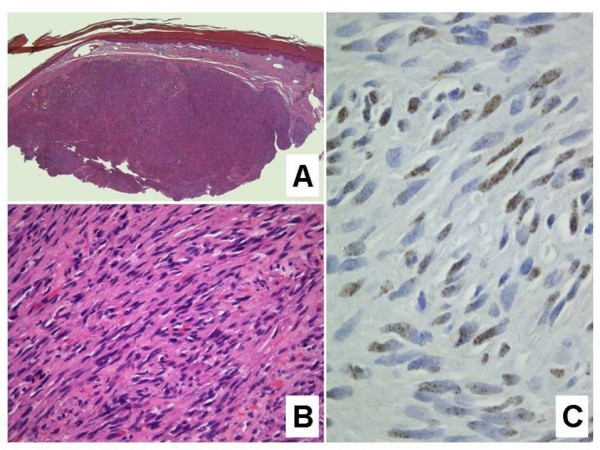
**Nodular Kaposi sarcoma.A**. The dermis is expanded by a solid tumor nodule (H&E stain). **B**. Fascicles of relatively monomorphic spindled cells, with slit-like vascular channels containing erythrocytes (H&E stain). **C**. The nuclei of the tumor cells demonstrate immunoreactivity for HHV-8 (LNA-1 immunohistochemical stain).

#### Nodular stage

**Figure 4:**
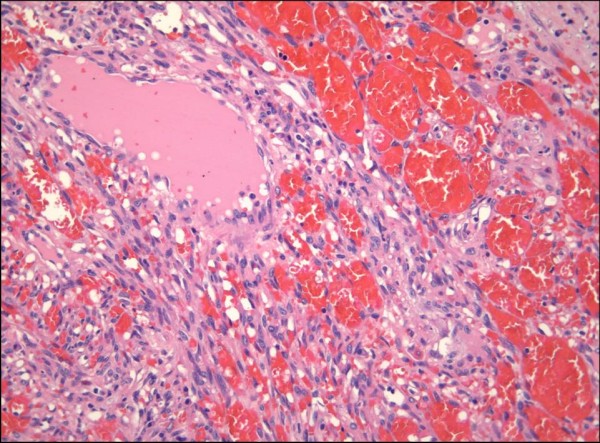
Nodular Kaposi sarcoma showing a peripheral component at higher magnification reminiscent of a cavernous hemangioma (H&E stain).

The nodular form of KS usually poses no diagnostic difficulties. Occasionally, however, a small ulcerated nodular KS lesion may be mistaken for a pyogenic granuloma [1]. Nodular KS exhibits dermal expansion by a relatively circumscribed, variable cellular proliferation of neoplastic spindled cells arranged in fascicles (Figure 4) [1-4]. Erythrocytes are contained within slit-like channels between the individual spindled cells. Although careful inspection may reveal occasional mitoses, the lesional cells are relatively monomorphic. Hyaline globules are seen more readily, as is the phenomenon of autolumination. In larger punch biopsy or excision biopsy specimens, the dermis away from the tumor nodule frequently exhibits changes associated with plaque stage KS, thus supporting the notion that patch, plaque and nodular stage lesions form part of a morphologic continuum. The periphery of some nodular KS lesions may show more dilated vascular spaces, imparting a pattern that is strikingly reminiscent of a cavernous hemangioma (Figure 4) [2]. These larger, congested channels are an integral part of the lesion, as confirmed by positive immunohistochemical staining of the lining endothelial nuclei for HHV-8 latent nuclear antigen 1 (LNA-1).

Large cutaneous nodules may frequently undergo ulceration. Superficial shave biopsies of such lesions may be diagnostically challenging to the histopathologist, as most of the specimen may contain only an inflammatory exudate with underlying granulation tissue; this may be misinterpreted as a pyogenic granuloma [1]. Distinguishing between spindle cells from granulation tissue and lesional KS cells from the uppermost portion of an underlying KS nodule can be difficult, if not impossible without the aid of immunohistochemistry. The commercial antibody to HHV-8 LNA-1 and the lymphatic endothelial cell marker D2-40 may prove very useful in this context. Staining with these markers is preferable to less specific vascular markers such as CD31 or CD34, as these do not facilitate recognition of the lesional and non-lesional endothelial cell populations. Rare instances of acquired immune deficiency syndrome (AIDS)-associated KS harboring a concomitant opportunistic pathogen (*e.g. *cryptococcosis) may also go undiagnosed in superficial biopsy material [14,15]. Superficial shave biopsies, therefore, should be discouraged.

Lesions that may potentially be confused histologically with nodular KS include bacillary angiomatosis, other vascular tumors (e.g. spindle cell hemangioma and Kaposiform hemangioendothelioma), fibrohistiocytic tumors (e.g. cellular, angiomatoid and atypical variants of fibrous histiocytoma, and dermatofibrosarcoma protuberans), resolving dermal fasciitis, spindle cell melanoma, and several other spindle cell mesenchymal neoplasms (e.g. cutaneous leiomyosarcoma) [1,2,4].

### Variants reported in the older literature

#### Anaplastic Kaposi sarcoma

Anaplastic KS, sometimes referred to as pleomorphic KS, is poorly documented in the literature, possibly because of its rarity. Malignant transformation of KS, characterized by an increase in the number of mitoses and marked cellular pleomorphism, was first described in 1959 by Cox and Helwig [16]. A "monomorphic" variant was identified by Templeton in several cases of African KS [17]. In a review of KS cases from Uganda (in 1971), investigators distinguished KS with a "monocellular pattern" (resembling anaplastic KS) from a so-called "anaplastic variant pattern" (resembling angiosarcoma) [18]. Anaplastic histology has been described in the context of classic, African, and AIDS-associated KS [7,8,19-24]. We are unaware of a report of this rare variant following iatrogenic immunosuppression.

**Figure 5:**
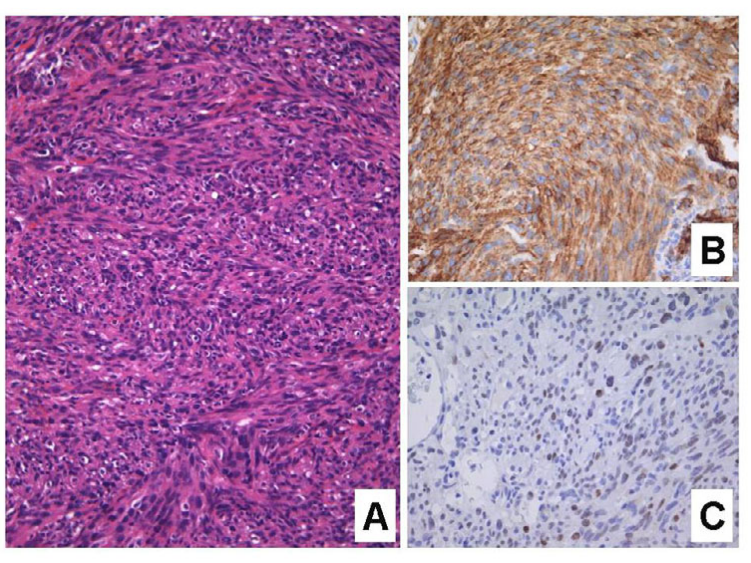
**Anaplastic Kaposi sarcoma. A**. Solid, haphazard proliferation of atypical spindled cells, with little evidence to suggest a vasoformative tumor (H&E stain). **B**. CD31 immunostain confirming the presence of a solid proliferation of atypical endothelial cells. **C**. LNA-1 immunostain for HHV-8 demonstrating positive immunoreactivity in the nuclei of several pleomorphic lesional cells.

Anaplastic KS is clinically notable for its high local aggressiveness, propensity for deep invasion, and increased metastatic capacity. Progressive histologic dedifferentiation in otherwise typical cases of KS has been noted (Figure pending permission) [22,25]. AIDS-associated tumors with anaplastic histology appear to have a propensity to occur in acral locations. In anaplastic KS, there is inherent potential for misdiagnosis as the vasoformative nature of the solid and frequently fascicular spindle cell proliferation is not readily apparent. This variant displays a significantly greater degree of nuclear and cellular pleomorphism than conventional nodular KS (Figure 5). In addition, there is an increased mitotic index (e.g. 5–20 mitoses per 10 high-power fields), and atypical mitoses may be encountered. Necrosis is occasionally observed.

It is easy to appreciate why a host of other malignant spindle cell neoplasms might be entertained in the histologic differential diagnosis, including certain sarcomas (e.g. leiomyosarcoma, spindle cell rhabdomyosarcoma, malignant peripheral nerve sheath tumor, fibrosarcoma), amelanotic spindle cell melanoma, and spindle cell carcinoma [14,15,20]. Angiosarcoma might also be considered, particularly if erythrocytes are identified between the markedly atypical spindled cells. A comprehensive panel of immunohistochemical stains is often required to rule out the aforementioned entities and confirm the presence of KS [15]. It is plausible that a proportion of anaplastic KS cases reported in the earlier literature, prior to the advent of immunohistochemistry, might not have been true cases of KS after all.

#### Lymphedematous variants

There are several variants of lymphedematous KS, all of which can present clinically with a deceptive bulla-like appearance (Figures pending permission). The interchangeable terminology that has been used in the literature for these variants is confusing. An attempt to classify this issue has been made [26], and includes variants associated with ectatic lymphatics such as lymphangioma-like and lymphangiectactic KS, and/or due to the accumulation of superficial dermal edema such as the subepidermal and intraepidermal (lymphatic) bullous KS variants. Most of these variants usually contain an admixture of more stereotypical KS lesions. In those cases where these lymphedematous variants form the predominant or sole histological pattern, the diagnosis of KS may be problematic.

##### Lymphangioma-like KS

Lymphangioma-like KS (LLKS), also referred to as "lympangiomatous" KS, is an uncommon variant that may be encountered in all four major clinicopathologic groups of KS patients [27-33]. Furthermore, lymphangioma-like morphology can occur in patch, plaque or nodular stage lesions [26]. This variant is said to account for less than 5% of KS cases [29]. Although Ronchese and Kern in 1957 are often credited with first describing the condition, the first reported case actually appears to date back to 1923, noted in a 66-year-old woman with clinical bullous KS lesions, the histology of which was described as being analogous to lymphangioma circumscriptum [27,34]. LLKS is most likely related to lymphedematous KS, bullous KS and hyperkeratotic (or verrucous) KS, as many of the reported patients with these clinical variants of KS have shown histopathologic features of LLKS on skin biopsy [10,26,27,34-39]. In some case reports patients with LLKS manifested with widespread and pronounced lymphedema [40] as well as effusions [41].

**Figure 6:**
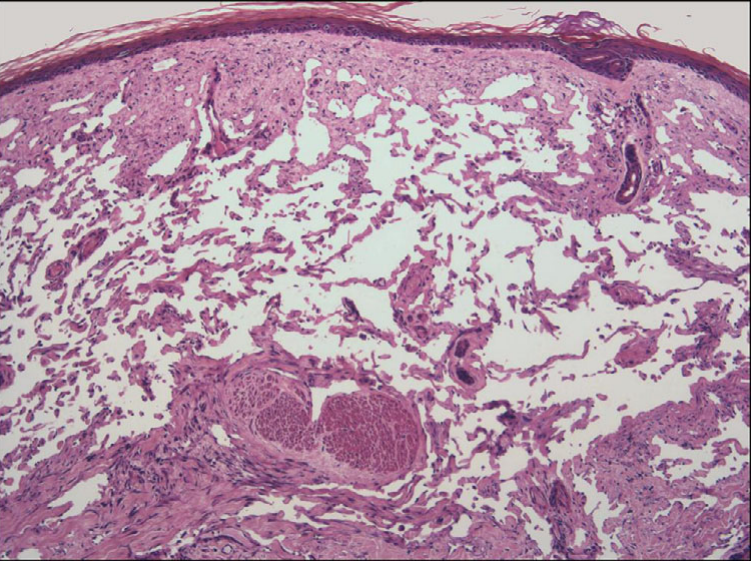
**Lymphangioma-like Kaposi sarcoma.** In this florid example, the dermis is replaced by a haphazard proliferation of gaping, interanastomosing channels splaying apart the dermal collagen (H&E stain).

**Figure 7:**
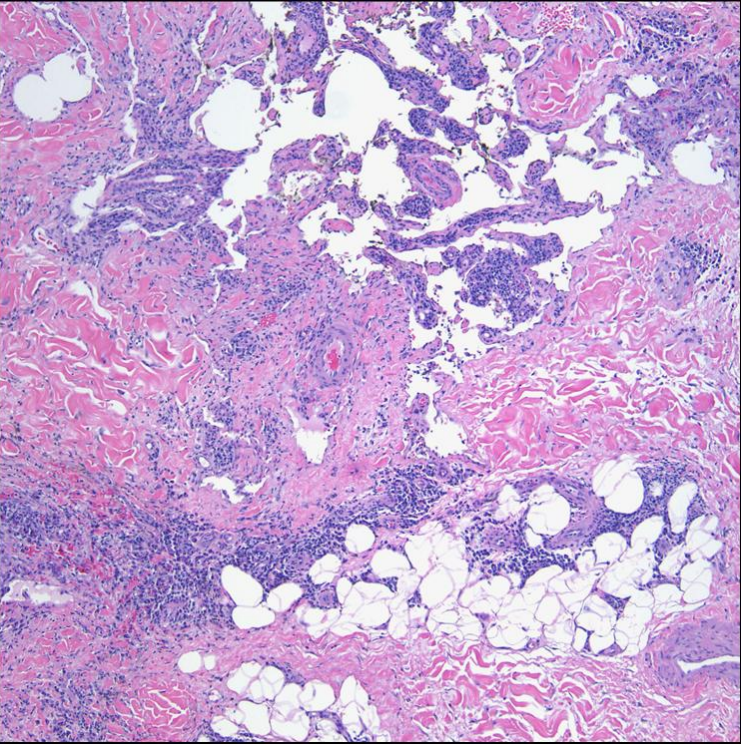
Lymphangioma-like Kaposi sarcoma seen at higher magnification in which the promontory sign is well demonstrated (H&E stain).

**Figure 8:**
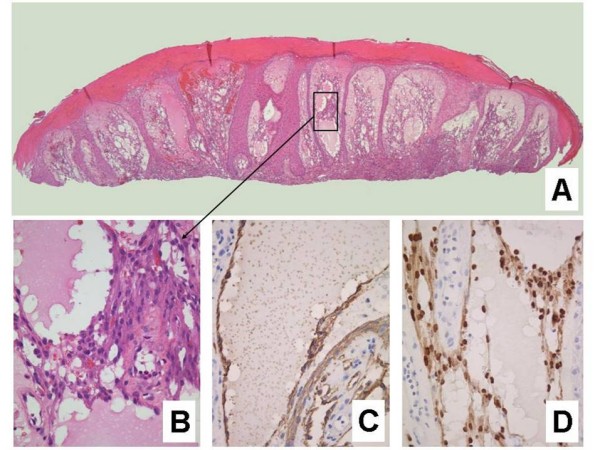
**Lymphangioma-like Kaposi sarcoma**. **A**. Low-power photomicrograph depicting the upper dermal expansion by a proliferation that is distinctly reminiscent of a lymphatic neoplasm or malformation. Note the reactive epidermal hyperplasia and hyperkeratosis (H&E stain). **B**. Closer view of the "lymphangiomatous" proliferation, with each gaping channel lined by a monolayer of plump Kaposi sarcoma cells, and frothy, pale eosinophilic intraluminal fluid resembling lymph (H&E stain). **C**. Positive staining of lining cells with the lymphatic endothelial marker D2-40. **D**. Nuclei of the same cellular population show immunoreactivity for LNA-1.

LLKS appears to exist (or co-exist) in two forms microscopically. The first comprises a patch or plaque stage lesion in which irregular, ectatic, interanastomosing vascular channels dissect dermal collagen bundles, resulting in a striking histological resemblance to a lymphatic tumor, such as a benign lymphangioendothelioma/acquired progressive lymphangioma (Figure 6) [31,32,42]. In these cases the promontory sign tends to be particularly conspicuous (Figure 7). Erythrocytes are usually absent from these channels. Slender papillae may project into vessels. In the second form, much larger, well formed endothelial lined spaces occupy the papillary dermis and the upper reticular dermis (Figure 8). These channels may closely abut on the overlying epidermis, in a pattern somewhat analogous to lymphangioma circumscriptum. It is the latter pattern which may give rise to the clinical appearance of "bullous" cutaneous lesions. Features of usual plaque stage KS are often encountered subjacent to these large channels; this useful diagnostic clue may be absent from biopsies that are too superficial, especially shave biopsies. The endothelial cells lining the ectatic, lymphangioma-like channels in both forms are immunoreactive for HHV-8 LNA-1, as well as the lymphatic endothelial marker D2-40 (Figure 8).

##### Lymphangiectatic Kaposi sarcoma

**Figure 9:**
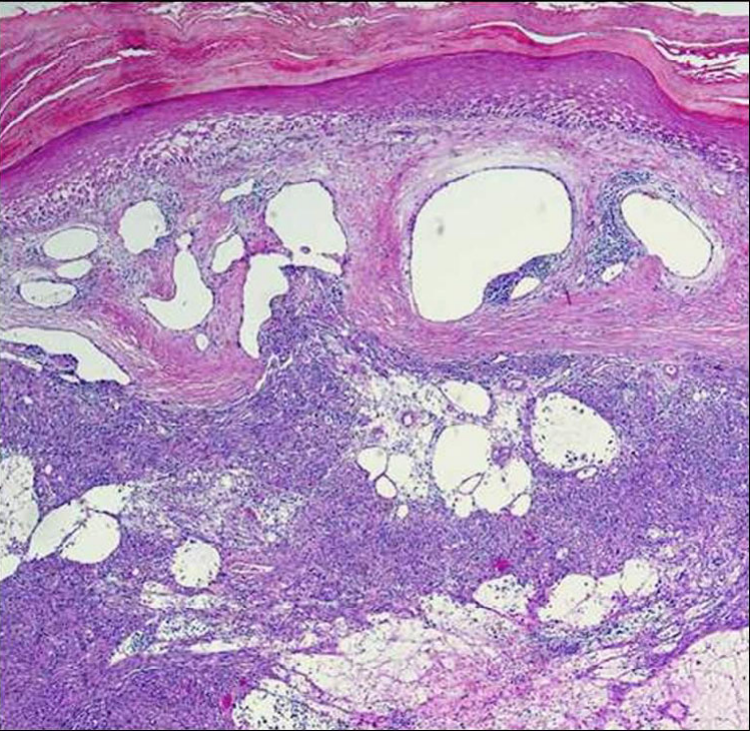
**Lymphangiectactic Kaposi sarcoma**. Large ecstatic lymphatics can be seen within and around this KS tumor nodule (H&E stain).

In lymphangiectactic KS there are large intratumoral and peritumoral dilated thin-walled lymphatic vessels (Figure 9). These ectatic lymphatics are much larger than those seen in LLKS, and less irregular and anastomosing [26]. They appear to be far less "compressible". Marked lymphangiectasia present in the superficial dermis may result in a bullous appearing lesion (pseudoblister).

##### Bullous Kaposi sarcoma

**Figure 10:**
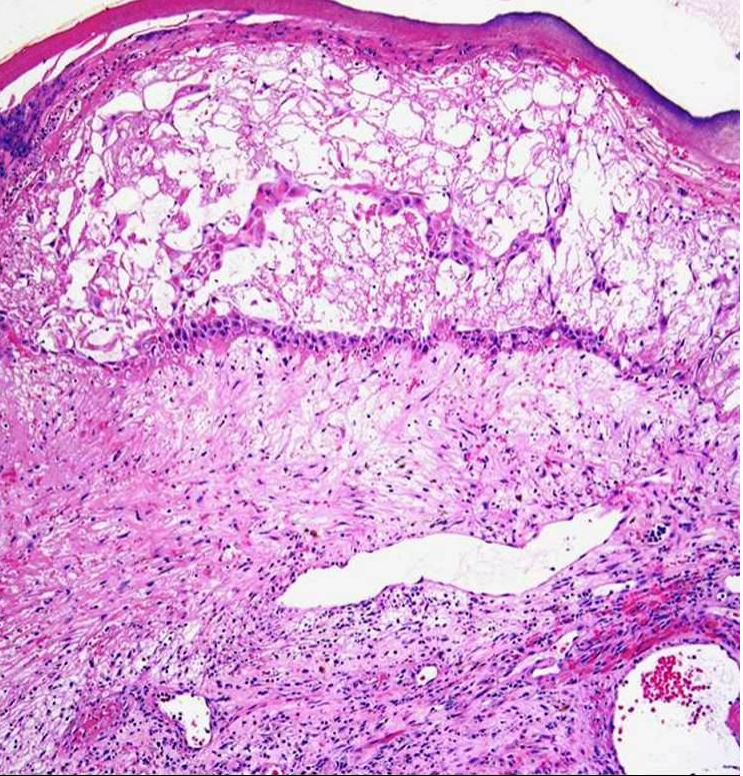
**Bullous Kaposi sarcoma**. In this patient with African endemic KS there is an intraepidermal bullous overlying subepidermal lymphedema associated with an underlying KS tumor nodule (not shown in this field) (H&E stain).

The first published descriptions of bullous cutaneous lesions in patients with KS appeared in the early part of the twentieth century. These bullous lesions were ascribed to lymphangiectases [34]. Bullous lesions are observed most frequently in the context of lymphedematous KS, but this is not always the case [26,34]. In most instances, the term "bullous" is clinical rather than pathologic, since pseudoblisters also occur as a consequence of lymphangiectasia and/or LLKS involving the superficial dermis in these patients (Figure 10) [26]. On other occasions, however, true subepidermal or intraepidermal bullae may arise in concert with KS. In the former, tense bullae are observed clinically due to peritumoral edema in the superficial dermis, while the latter may evolve either as a result of progression of a subepidermal bulla or due to resorption of lymphedema and re-epithelialization of a subepidermal blister [26].

#### Telangiectatic KS

**Figure 11:**
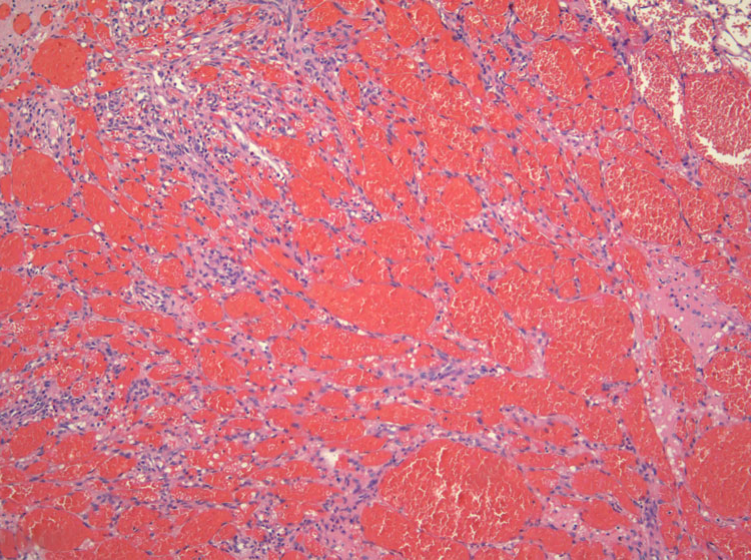
**Telangiectatic Kaposi sarcoma is characterized by intensely congested, ectatic vascular spaces lined by lesional cells (H&E stain)**.

**Figure 12:**
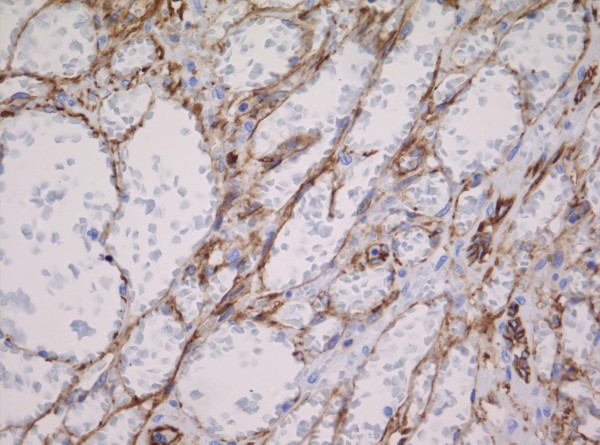
A CD31 immunostain highlights the many dilated vascular spaces seen in telangiectatic Kaposi sarcoma.

There is a single case report of telangiectatic KS, which occurred in a man with thymoma and myasthenia gravis receiving long-term immunosuppressive therapy [43]. The term "telangiectatic" referred to the significant telangiectasia associated with the multiple cutaneous nodules, and not the histopathologic features thereof. The histopathology in this case report showed usual features of nodular KS, with no conspicuous background vascular ectasia [43]. The authors have encountered rare histological examples of telangiectatic KS in which nodular KS lesions contained large, intensely congested, ectatic vascular spaces (Figure 11). Since these large spaces are lined by endothelial cells (Figure 12) whose nuclei are immunoreactive for LNA-1, it must be assumed that they are an integral part of the KS and not merely native dermal vessels that have undergone telangiectasia as a consequence of compression by the dermal tumor.

### Contemporary variants

#### Hyperkeratotic (Verrucous) Kaposi sarcoma

**Figure 13:**
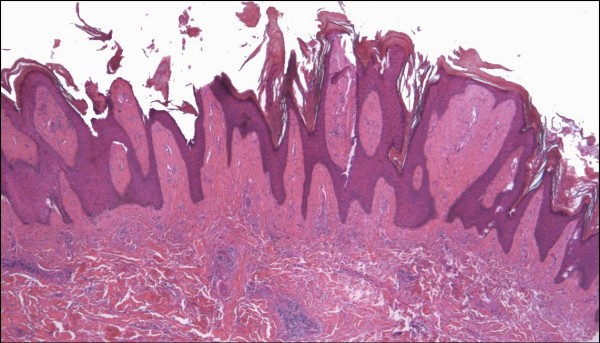
**Hyperkeratotic (verrucous) Kaposi sarcoma**. A plaque stage lesion from the lower leg is surfaced by an epidermis showing verruciform acanthosis and hyperkeratosis, with fibrosis of the upper dermis (H&E stain).

**Figure 14:**
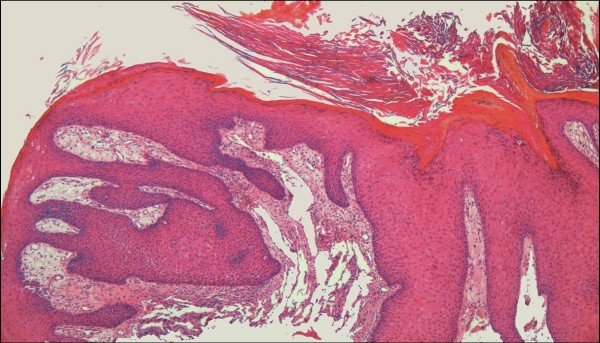
**Kaposi sarcoma with hyperkeratotic and lymphangioma-like histologic features (H&E stain)**. Note the dilated channels in the dermis and the marked acanthosis, and hyperkeratosis of the overlying epidermis.

Hyperkeratotic KS is a rarely described clinicopathalogic variant of KS, which appears to be closely linked to severe KS-associated lymphedema in patients with AIDS [10,35,44]. There is verrucous epidermal acanthosis and hyperkeratosis overlying an often fibrotic epidermis (Figure 13). In view of the latter feature, diagnostic KS lesional tissue may be located at a relatively deeper level in the dermis, further emphasizing the potential inadequacy of superficial shave biopsies. On occasion, verrucoid epidermal changes may occur with LLKS histology (Figure 14). Infrequently, such changes may involve the entire lower extremity manifesting as elephantiasis nostras verrucosa [45]. Chronic lymphedema may itself give rise to verruciform epidermal hyperplasia and hyperkeratosis, with increased fibroblastic activity, blood vessels and thick-walled lymphatic vessels throughout the dermis [39]. Lympedematous AIDS-associated KS may also be associated with exophytic fibroma-like nodules, characterized by dermal fibrosis, a loose arrangement of fibroblasts and collagen bundles, and dilated blood vessels and lymphatic channels [10,39,44].

#### Keloidal Kaposi sarcoma

**Figure 15:**
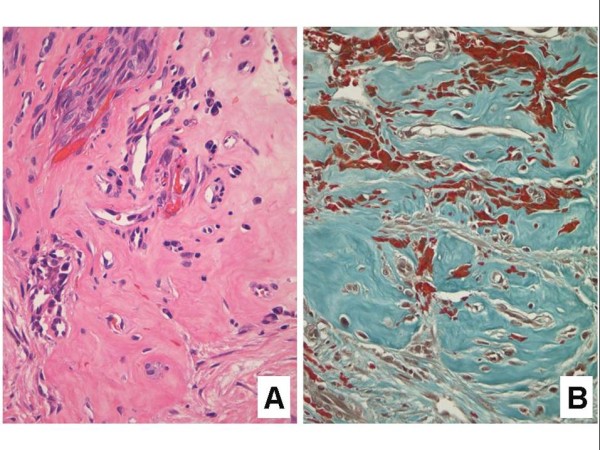
**Keloidal Kaposi sarcoma.A**. Spindled cells from the edge of the Kaposi sarcoma plaque lesion (upper left) are flanked by an expanse of keloid-like collagen (lower right) (H&E stain). **B**. Masson's trichrome stain highlighting the keloidal collagen bundles. Note the many extravasated erythrocytes in the background.

The description of this exceedingly uncommon KS variant is limited to a 1994 report of three cases [46]. Lesions are firm and rubbery, and may be linear [6]. Histologically, there is notable dermal expansion by dense, hyalinized collagen with a distinct resemblance to a keloid (Figure 15). In such lesions the spindled KS proliferation may be obscured by these keloidal alterations. The histologic differential diagnosis includes a dermal scar at the site of a previous skin biopsy of a KS lesion. It is postulated that cytokines play a key role in the evolution of the keloidal stromal changes in this unusual variant [46].

#### Micronodular KS

**Figure 16:**
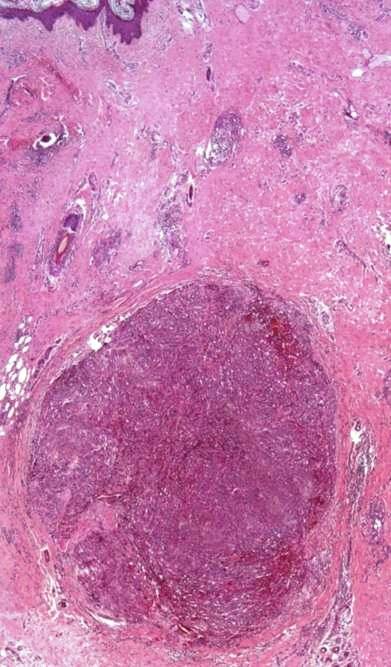
**Micronodular Kaposi sarcoma.** A small, solid circumscribed spindle cell proliferation is present in the mid- to lower dermis (H&E stain).

Micronodular KS (Figure 16) is a recently described variant of nodular KS, which is characterized histologically by a small, unencapsulated, circumscribed spindle cell proliferation in the reticular dermis [47]. Although the paper by Kempf et al described micronodular cutaneous lesions in a patient with classic KS [47], similar lesions are occasionally encountered in the context of AIDS-associated KS, and are often removed in their entirety by a punch biopsy.

#### Pyogenic granuloma-like Kaposi sarcoma

**Figure 17:**
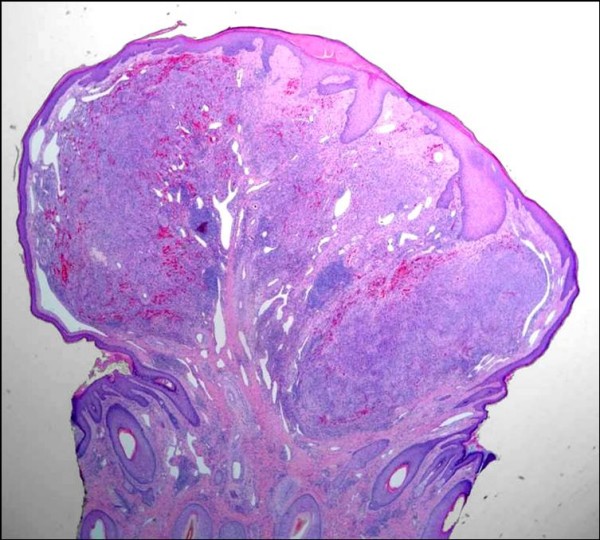
Pyogenic granuloma-like Kaposi sarcoma manifesting as an exophytic mass enveloped by an epidermal collarette (H&E stain).

Small, superficially located nodular or micronodular KS lesions may be protuberant and thereby elicit the development of a peripheral epidermal collarette (Figure 17). Such lesions have been referred to as pyogenic granuloma (PG)-like KS [48]. Traumatized lesions may undergo ulceration and become inflamed, and potentially misdiagnosed as a true PG (lobular capillary hemangioma). To further complicate matters true PGs may themselves harbor kaposiform areas. PG-like KS must also be distinguished from bacillary angiomatosis, as some examples of the latter can adopt striking PG-like low-power architecture [14,15].

#### Ecchymotic Kaposi sarcoma

**Figure 18:**
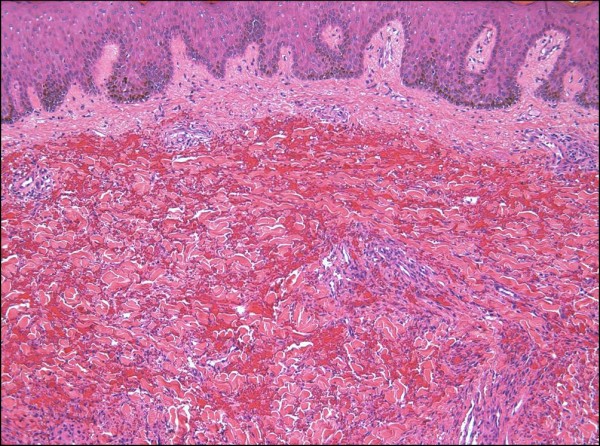
**Ecchymotic Kaposi sarcoma.** The spindled cell proliferation in this example is somewhat obscured by the extensive purpura.

In the variant referred to as ecchymotic KS, the intradermal KS proliferation is accompanied by extensive red blood cell extravasation (Figure 18) [49]. The marked purpura often obscures the underlying histologic features of KS. The differential diagnosis includes intralesional hemorrhage brought about the biopsy procedure itself. Clinically, this variant of AIDS-associated KS manifests with ecchymotic or pityriasis-like patches [49]. Ecchymotic plaque lesions may clinically also resemble a bruise or port-wine stain. A rare case of clinical "hemorrhagic" KS has been reported [50]. However, it is unclear if the appearance in this case was attributed to extensive erythrocyte extravassation.

#### Intravascular Kaposi sarcoma

The only description of intravascular KS is limited to a report of six cases, including four patients with classic KS and two with AIDS-associated KS [11]. Histologic examination in this small series showed an exclusively intravascular solid spindle cell KS proliferation. Immunohistochemical stains for desmin and smooth muscle actin (SMA) confirmed that this proliferation was indeed intravenous. The histologic differential diagnosis includes intravascular papillary endothelial hyperplasia, intravenous PG, intravascular fasciitis, papillary intralymphatic angioendothelioma (Dabska tumor) and intravascular myopericytoma [51].

#### Variants related to therapy

Therapy may result in KS regression, and less likely exacerbation (so-called KS flare) [52]. The histopathology of regression in KS has been previously described and is discussed below. KS exacerbation (flare or recrudescence) can occur following therapy with corticosteroids, after treatment with rituximab, or as part of the immune reconstitution inflammatory syndrome (IRIS) seen with antiretroviral therapy in HIV-infected persons [52]. The histomorphology of KS flare lesions has yet to be described.

##### Regressing Kaposi sarcoma

**Figure 19:**
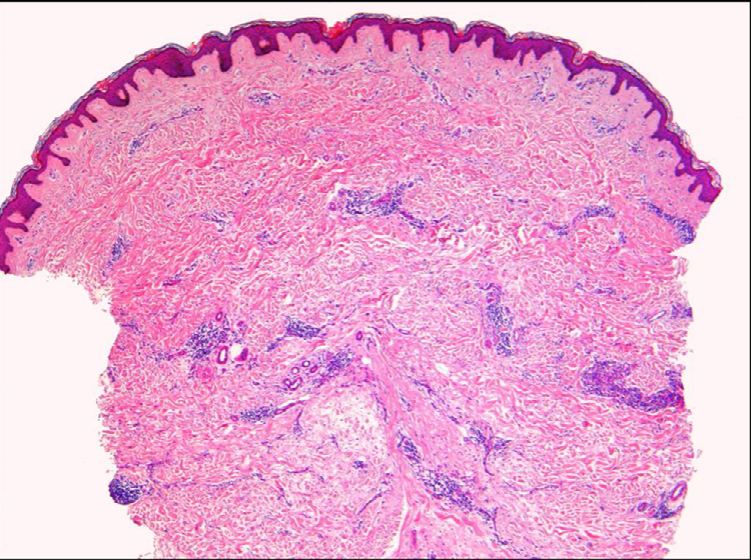
**Regressed Kaposi sarcoma lesion**. There is a noticeable increase in dermal capillary density with an associated lymphoplasmacytic infiltrate around native dermal vessels and appendages (H&E stain).

**Figure 20:**
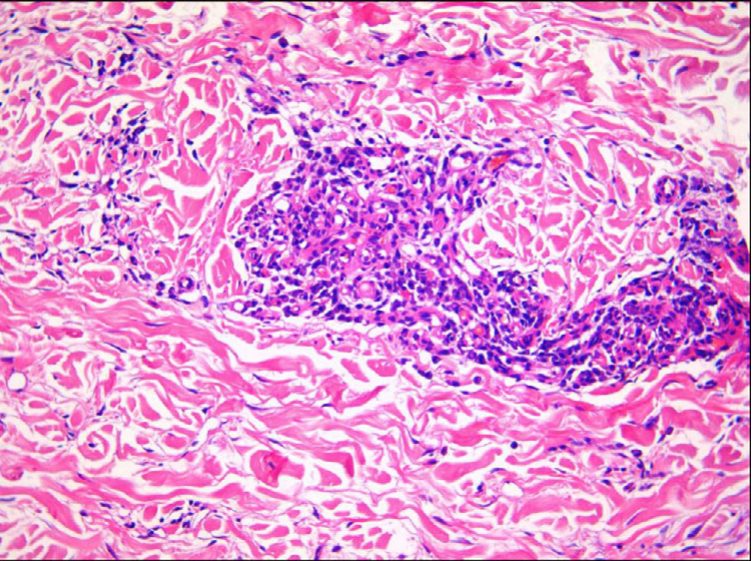
Regressed Kaposi sarcoma lesion seen at higher magnification showing a perivascular infiltrate comprised mainly of plasma cells (H&E stain).

**Figure 21:**
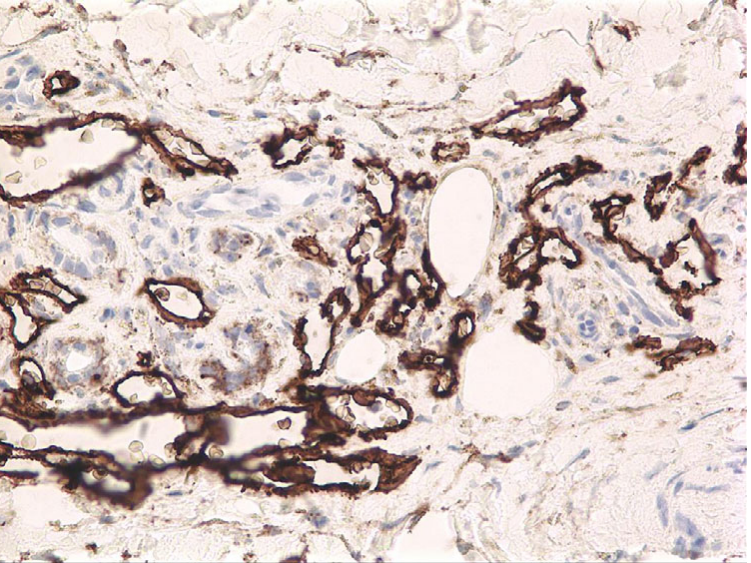
**A completely regressed Kaposi sarcoma lesion still retains a modest amount of abnormal dermal microvessels, as evidenced by this D2-40 immunostain.** D2-40 is a marker of lymphatic endothelium.

The introduction of highly active antiretroviral therapy (HAART) for patients with human immunodeficiency virus infection (HIV) may lead to complete regression of established AIDS-associated KS lesions [53,54]. Clinical features of regression include flattening of lesions, reduction in lesion size, and change from a purple-red appearance to an orange-brown macule. Following antiretroviral therapy, investigators have observed improved circumscription of nodular lesions, which appear less cellular and are enveloped by a densely sclerotic stroma [55]. In some cases the only significant abnormalities are an increase in dermal capillary density around native dermal vessels and appendages (Figure 19), and an accompanying perivascular infiltrate of largely plasma cells (Figure 20). Partial or complete regression of KS lesions may also be brought about following the administration of chemotherapeutic agents [56,57]. Histologic examination of such partially regressed lesions reveals residual spindled cells around native vessels in the mid- and upper dermis, and a significant reduction in the number of spindled lesional cells in the intervening dermis [56]. Lesions that have undergone complete regression, however, show an absence of these spindled cells, and a modest increase in microvessels (Figure 21) in relation to the superficial vascular plexus [56]. Other findings include the presence of hemosiderin-laden dermal macrophages and a conspicuous superficial perivascular lymphocytic infiltrate [56].

## Conclusion

KS clearly has the ability to develop into lesions of varying morphologic appearance. It is important to be able recognize these variants in order to avoid potential misdiagnosis and improper management of afflicted patients. KS has been shown to be of lymphatic origin [58]. This may explain the intimate association of abnormal lymphatics observed in several of these variants, such as lymphangioma-like and lymphangiectatic KS. KS is also intimately associated with lymphedema. Chronic lymphedema may even precede KS lesions. Some authors believe that chronic lymphedema may promote KS development due to a combination of collateral vessel formation, lympahngiogenesis and immune impairment [59]. Hyperkeratotic and bullous KS variants can be attributed to the long standing effects of tumor-associated lymphedema on the overlying epidermis. Deep dermal fibroma-like nodules seen in cases associated with marked lymphedema could explain the origin of micronodular KS. The clinical significance of most of these KS variants has not been studied. Lymphedematous variants have been postulated to portend a poor prognosis. This is certainly plausible given the fact that significant KS-related edema carries a grave prognosis [60]. Anaplastic KS is perhaps the only variant associated with aggressive behaviour. The reason for progressive histological dedifferentiation in some cases of KS is unknown. Studies looking at the role of HHV-8 and the host (e.g. immunity) in anaplastic cases may provide some answers.

## Competing interests

The authors declare that they have no competing interests.

## Authors' contributions

LP and WG contributed equally to the literature review, photography of cases, and writing of this manuscript. Both authors read and approved the final manuscript.
